# Development of a Scorecard to Monitor Progress toward National Cholera Elimination: Its Application in Uganda

**DOI:** 10.4269/ajtmh.23-0007

**Published:** 2023-04-10

**Authors:** Godfrey Bwire, David A. Sack, Stella M. Lunkuse, Francis Ongole, Moise Chi Ngwa, Didacus B. Namanya, Jesca Nsungwa, Jane Ruth Aceng Ocero, Henry G. Mwebesa, Allan Muruta, Anne Nakinsige, Annet Kisakye, Peter Kalyebi, Julian Kemirembe, Issa Makumbi, Atek Kagirita, Immaculate Ampeire, David Mutegeki, David Matseketse, Amanda Kay Debes, Christopher Garimoi Orach

**Affiliations:** 1 Department of Community Health, Ministry of Health Uganda, Kampala, Uganda;; 2 Department of International Health, Johns Hopkins Bloomberg School of Public Health, Baltimore, Maryland;; 3 Division of Surveillance, Knowledge and Information Management, Ministry of Health, Kampala, Uganda;; 4 Department of National Health Laboratory and Diagnostic Services, Ministry of Health, Kampala, Uganda;; 5 Department of Environmental Health, Ministry of Health, Kampala, Uganda;; 6 Department of Maternal and Child Health, Ministry of Health, Kampala, Uganda;; 7 Office of the Minister, Ministry of Health, Kampala, Uganda;; 8 Office of the Director General Health Service, Ministry of Health, Kampala, Uganda;; 9 Department of Integrated Epidemiology and Public Health Emergencies, Ministry of Health, Kampala, Uganda;; 10 Division of Public Health Emergency Preparedness and Response, Ministry of Health, Kampala, Uganda;; 11 World Health Organization, Kampala, Uganda;; 12 Masaka Regional Referral Hospital, Masaka City, Uganda;; 13 Public Health Emergency Operation Centre, Ministry of Health, Kampala, Uganda;; 14 Uganda National Immunization Programme, Ministry of Health, Kampala, Uganda;; 15 UNICEF, Kampala, Uganda;; 16 College of Health Sciences, Makerere University School of Public Health, Kampala, Uganda

## Abstract

In 2017, the Global Task Force for Cholera Control (GTFCC) set a goal to eliminate cholera from ≥ 20 countries and to reduce cholera deaths by 90% by 2030. Many countries have included oral cholera vaccine (OCV) in their cholera control plans. We felt that a simple, user-friendly monitoring tool would be useful to guide national progress toward cholera elimination. We reviewed cholera surveillance data of Uganda from 2015 to 2021 by date and district. We defined a district as having eliminated cholera if cholera was not reported in that district for at least 4 years. We prepared maps to show districts with cholera, districts that had eliminated it, and districts that had eliminated it but then “relapsed.” These maps were compared with districts where OCV was used and the hotspot map recommended by the GTFCC. Between 2018 and 2021, OCV was administered in 16 districts previously identified as hotspots. In 2018, cholera was reported during at least one of the four previous years from 36 of the 146 districts of Uganda. This number decreased to 18 districts by 2021. Cholera was deemed “eliminated” from four of these 18 districts but then “relapsed.” The cholera elimination scorecard effectively demonstrated national progress toward cholera elimination and identified districts where additional resources are needed to achieve elimination by 2030. Identification of the districts that have eliminated cholera and those that have relapsed will assist the national programs to focus on addressing the factors that result in elimination or relapse of cholera.

## INTRODUCTION

Cholera, a severe diarrheal disease caused by toxigenic *Vibrio cholerae* serotype O1, continues as a major public health threat in Africa and Asia.^1^ In 2017, the Global Task Force for Cholera Control (GTFCC) set a goal to eliminate cholera from ≥ 20 countries and to reduce cholera deaths by 90% by the year 2030.^2^ They outlined three “axes” in the GTFCC Roadmap to facilitate this goal, including 1) early detection and quick response to contain outbreaks; 2) a targeted prevention strategy in cholera hotspots, including the use of oral cholera vaccine; and 3) GTFCC support and coordination of human, technical, and financial resources.

The concept of elimination from many endemic countries is an ambitious goal given that cholera has continuously been reported in many sub-Saharan Africa countries since the early 1970s.^3^^–^^6^ Outside Africa, large outbreaks continue to be reported from widely scattered areas, including Yemen,^7^^,^^8^ Pakistan,^9^ as well as the Ganges Delta, which is considered the homeland of cholera.^10^ It was thought to have been eliminated from Haiti, where it caused many cases and deaths between 2010 and 2019^11^ but then reappeared in late 2022.^12^ Although there is variation in the numbers of cases reported to the WHO each year, there has not been a clear, long-term downward trend in the numbers of cases reported annually.

The GTFCC outlined a “Roadmap” for cholera elimination by 2030.^2^ The GTFCC also provides technical guidance and resources, including oral cholera vaccine (OCV) through the global stockpile funded by GAVI.^13^ Although GTFCC supports the partner states, the countries themselves must develop policies and implement prevention and control interventions based on an assessment of their national cholera burden. The GTFCC developed a tool to help countries identify cholera “hotspots” where vaccine and WaSH (water-sanitation-hygiene) interventions should be targeted.^14^ The GTFCC tool uses national cholera reports from the recent past (generally 5 years). This tool identifies districts that have high average annual rates of cholera, especially where cholera is reported persistently (high proportion of weeks reporting cholera). This hotspot analysis assists the countries to identify the districts within the country where control efforts should focus as they develop their national cholera control and elimination plans. The hotspot analysis has been especially useful for prioritizing areas for vaccine campaigns, but the intention is to also use this analysis for prioritizing WaSH interventions.

We thought that an additional tool was needed to assist the countries to monitor progress toward the national goal of cholera elimination. As a general guide, a country can be certified as having eliminated cholera if, despite excellent and sensitive surveillance, there are no cholera cases for three consecutive years. We reasoned that a similar approach could be used for determining if subnational areas (e.g., districts) had eliminated cholera, but to be more conservative, we thought that ≥ 4 years with no reported cases was more appropriate because these are smaller geographic areas. Many African countries are large geographically and have diverse populations living in different ecozones, and the districts within the country have different risks for cholera. In fact, as illustrated by the national hotspot maps of African countries,^15^^,^^16^ some districts have a much higher risk, and some districts have not reported cholera for many years.

**Figure 1.**
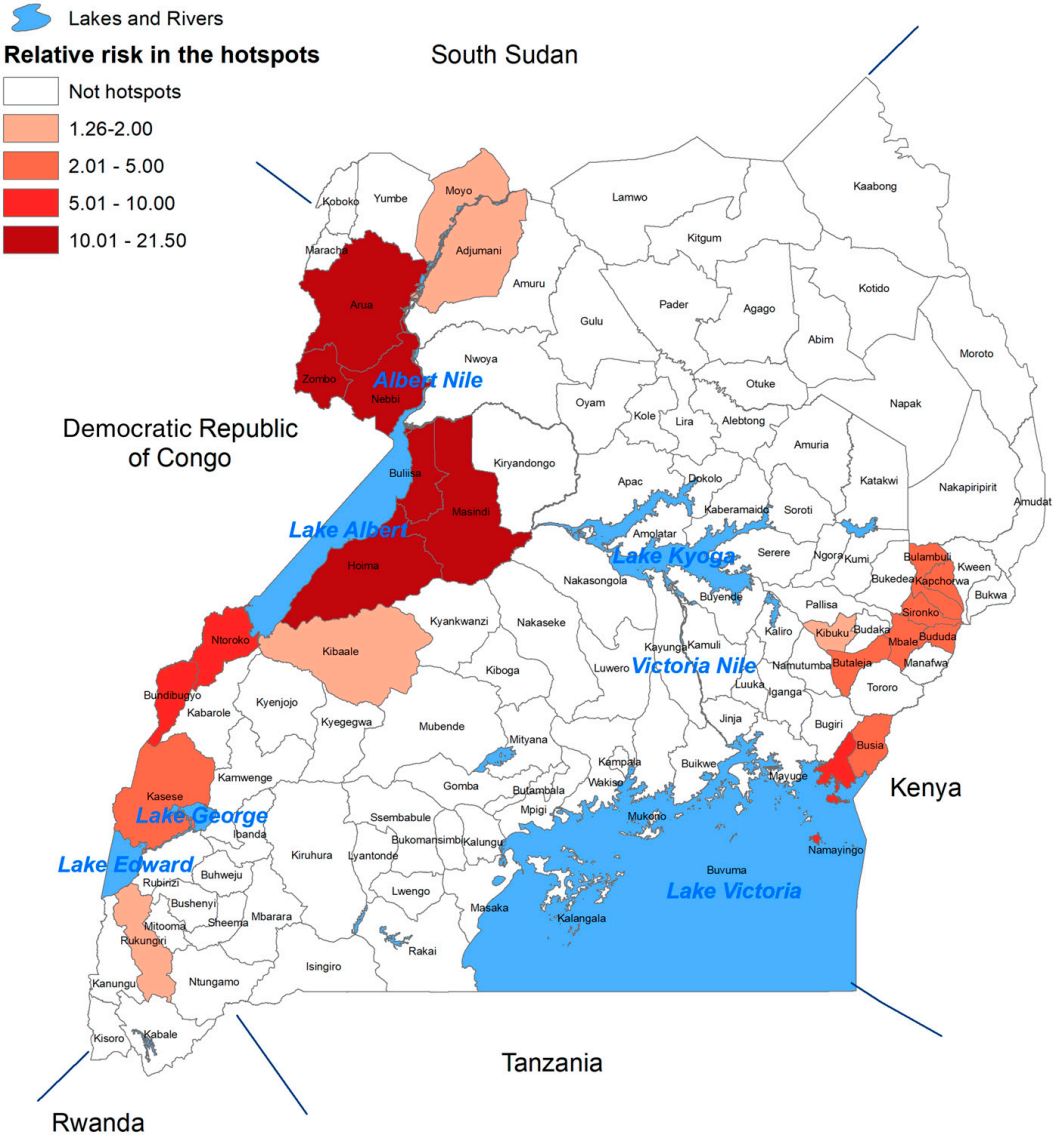
Map of Uganda showing cholera hotspots identified using data from 2011 to 2016.^16^ The SaTScan method identifies districts with a statistically significant increase in the risk of cholera compared with the country as a whole.

**Figure 2.**
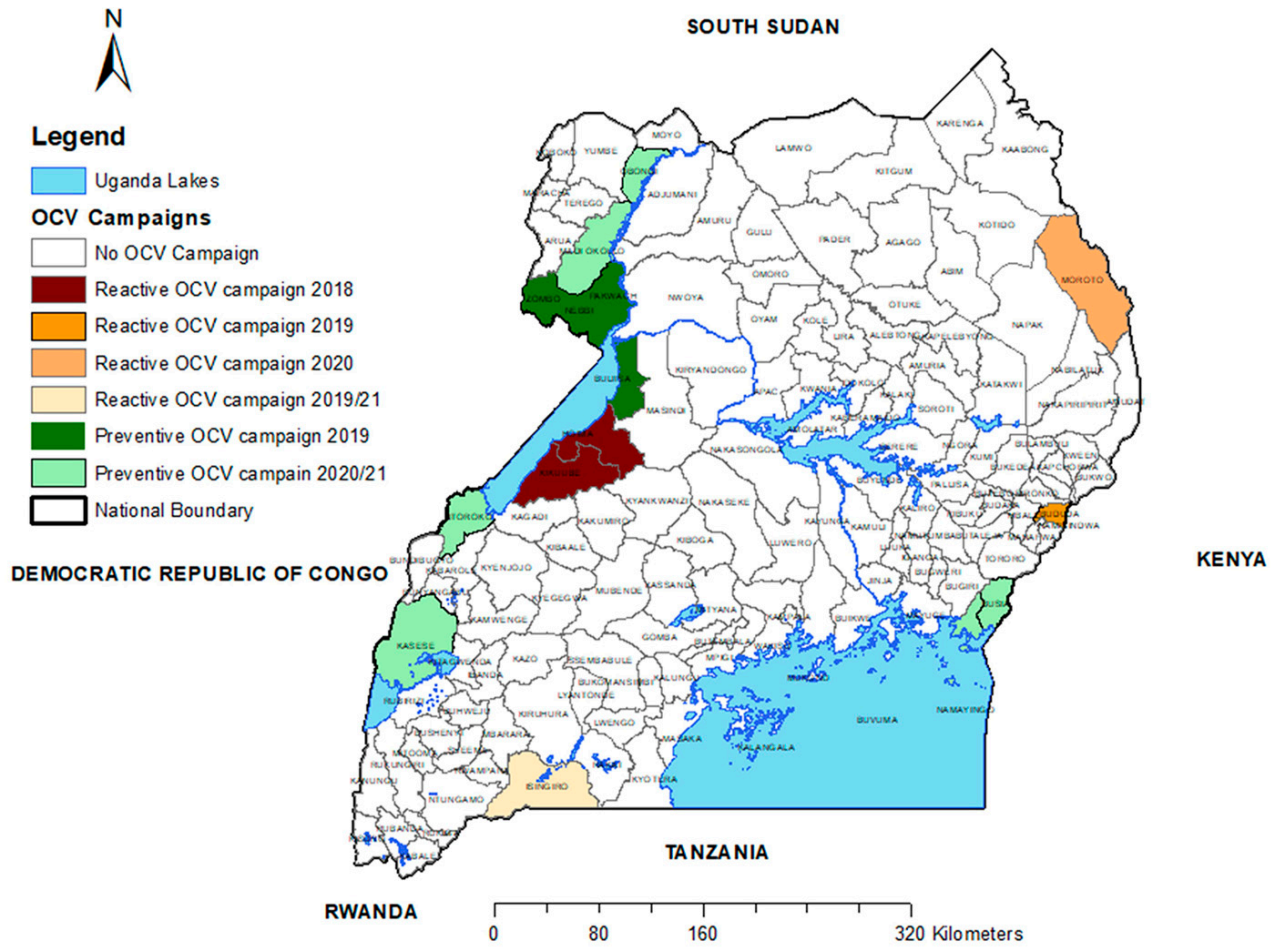
Map of Uganda showing districts where oral cholera vaccine (OCV) campaigns were conducted, 2018–2021.

In 2017, Uganda developed a cholera control plan, the National Integrated Comprehensive Cholera Prevention and Control Plan, Fiscal Years (2017/18–2021/22).^17^ Importantly, the Uganda Ministry of Health reviewed the cholera hotspot information during a national workshop in January 2018^18^ and, based on a hotspot analysis of cholera in Uganda, planned a series of OCV campaigns. Between 2018 and 2021, a total of 4,308,380 doses of OCV were administered during OCV campaigns in 16 districts. The previously published hotspot map and a map showing the locations and dates of the OCV campaigns are provided in Figures 1 and 2, respectively. Within the hotspot district, local knowledge was used to target the subcounties in the district as being ones at highest risk. Thus, the campaigns provided vaccine only to these higher-risk subcounties, not the entire district. Subjectively, these campaigns seemed to have reduced the number of cases and number of outbreaks in these districts where OCV was used, but a more formal analysis had not been carried out. The OCV campaign map was generated using data from OCV campaign reports for the period 2018–2021.

The current cholera control plan extended up to June 2022, and Uganda is now developing a new plan for the years up to 2030. We thought that a “cholera elimination scorecard” would be useful as Uganda develops its plans to update its cholera control plan, and such a scorecard may serve as a model for other countries to monitor progress toward national elimination by 2030. We intended the scorecard to illustrate districts in the country that reported cholera recurrently as well as districts that reported no cases for many years. We also wanted the scorecard to highlight districts that reported cholera after having been cholera free for many years. We also wanted to compare the maps generated by the scorecard map with the hotspot map as suggested by the GTFCC to understand the information each method provides.

## MATERIALS AND METHODS

This study was based on anonymous surveillance data collected by the Uganda Ministry of Health as part of the Health Management Information System, and the analysis was deemed to be not human subject’s research by the Johns Hopkins School of Public Health IRB.

### Data collection and analysis.

For each of the analyses, we used weekly cholera report data from the Ministry of Health, which included the number of cases in each district between 2015 and 2021. To calculate incidence, we used the population of each district, which is available from the Uganda Bureau of Statistics.^19^ Some districts were split into two districts during the time of analysis, which changed the geographical size and population of a few of the districts. Even when the districts were split, the geographical boundaries of the subcounties remained constant, and cases were reported by subcounty consistently.

To characterize cholera outbreaks in Uganda, weekly data from each district were analyzed to determine the beginning and end dates of each outbreak in the district. These data also provided the duration of the respective outbreaks. Clearly, an outbreak can spread between districts, but for the purpose of this analysis, we analyzed the cases occurring in each district separately. Based on a description of the outbreak in each district, we described the outbreaks in Uganda by the number of districts affected, the number of cases, the number of outbreaks, and the duration of outbreaks.

### Updating map of cholera hotspots.

Because the earlier hotspot map used data from 2011 to 2016,^16^ we developed a revised hotspot map. To generate the revised hotspot map, we used the method recommended by the GTFCC using data for the previous 7 years (2015–2021).^14^ Among the districts reporting cholera during this period, districts with a mean annual incidence higher than the 70^th^ percentile were considered high, and districts with persistence higher than the 70^th^ percentile were considered high. Districts with both high mean annual incidence and high persistence were given highest priority. Those with either high mean annual incidence or high persistence were considered moderate priority. Districts with cholera but with both lower mean annual incidence and low persistence were considered lower priority. Districts without any cholera reports during the period between 2015 and 2021 were considered to have eliminated cholera.

To generate the scorecard map, we used the annual district-specific data for the years 2015–2021. Beginning in 2018, we examined the data to determine if cholera had occurred in that year or any of the three previous years in that district, regardless of the number of cases occurring in that district. If no cases were reported in 2018 or in any of the three previous years, cholera was deemed to have been eliminated from that district. Similarly, data from each district were examined for the years 2019, 2020, and 2021 to determine if cholera was reported in that year or in the previous 3 years. If cholera was reported in any of these years in that district, the district was considered endemic, but if no cases were reported, cholera was deemed to be eliminated that year. If a district was determined to have eliminated cholera during one or more of the years between 2018 and 2020 and then again began reporting cholera, it was labeled as having “relapsed.”

## RESULTS

Among the 146 districts in Uganda, 41 districts reported cholera during the years between 2015 and 2021. A summary of these outbreaks is shown in Table 1. The other districts did not report any cases between 2015 and 2021 and were deemed to have eliminated cholera.

**Table 1:** Summary of characteristics of cholera outbreaks in Uganda 2015 to 2021

Characteristics	2015	2016	2017	2018	2019	2020	2021	Total
Number of districts with an outbreak	8	27	3	11	6	5	1	61*
Number of outbreaks	8	28	3	12	6	5	1	63
Number of cases	1,270	2,316	265	2,754	359	1,361	173	8,498
Duration of outbreaks, mean in weeks (SD)	8.6 (4.6)	8.6 (7.9)	5.3 (5.9)	7.8 (5.0)	8.8 (4.1)	7.6 (3.6)	5.00 (0.0)	8.2 (6.1)

* The districts that reported cholera outbreaks were Arua, Hoima, Kikuube, Kasese, Kampala, Busia, Mbale, Wakiso, Bulambuli, Pakwach, Nebbi, Zombo, Butaleja, Kibuku, Kapchorwa, Namayingo, Mayuge, Manafwa, Sironko, Buliisa, Kayunga, Adjumani, Yumbe, Amuru, Moroto, Buvuma, Pallisa, Namutumba, Madi Okollo, Budaka, Kisoro, Kyegegwa, Kagadi, Amudat, Kween, Tororo, Isingiro, Bududa, Kotido, Nabilatuk, and Napak.

**Figure 3.**
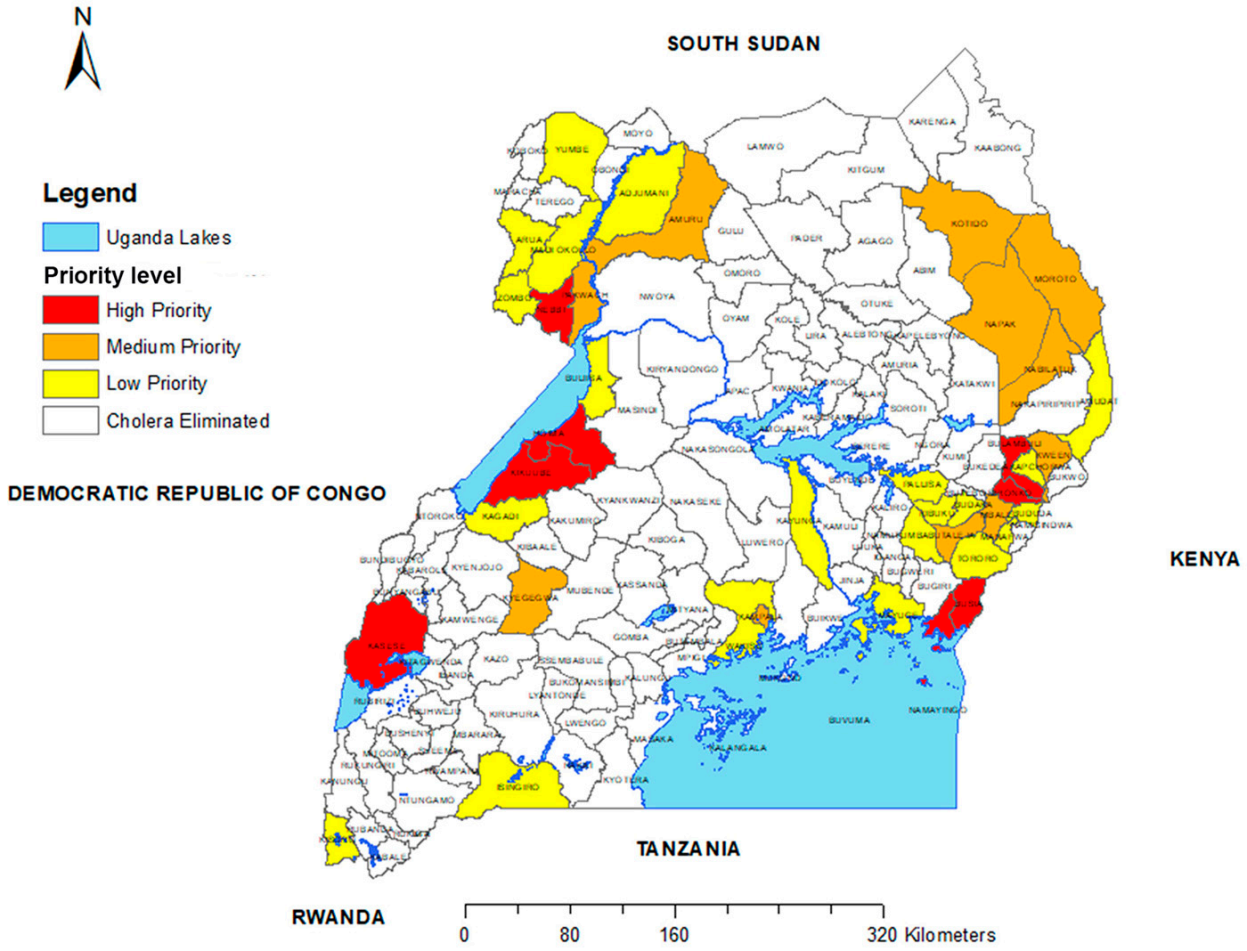
Uganda cholera hotspot map using the Global Task Force for Cholera Control method and using data from the years 2015–2021.

The current cholera hotspot map for Uganda using the GTFCC tool and the data of cholera reports between 2015 and 2021 is shown in Figure 3.

**Figure 4.**
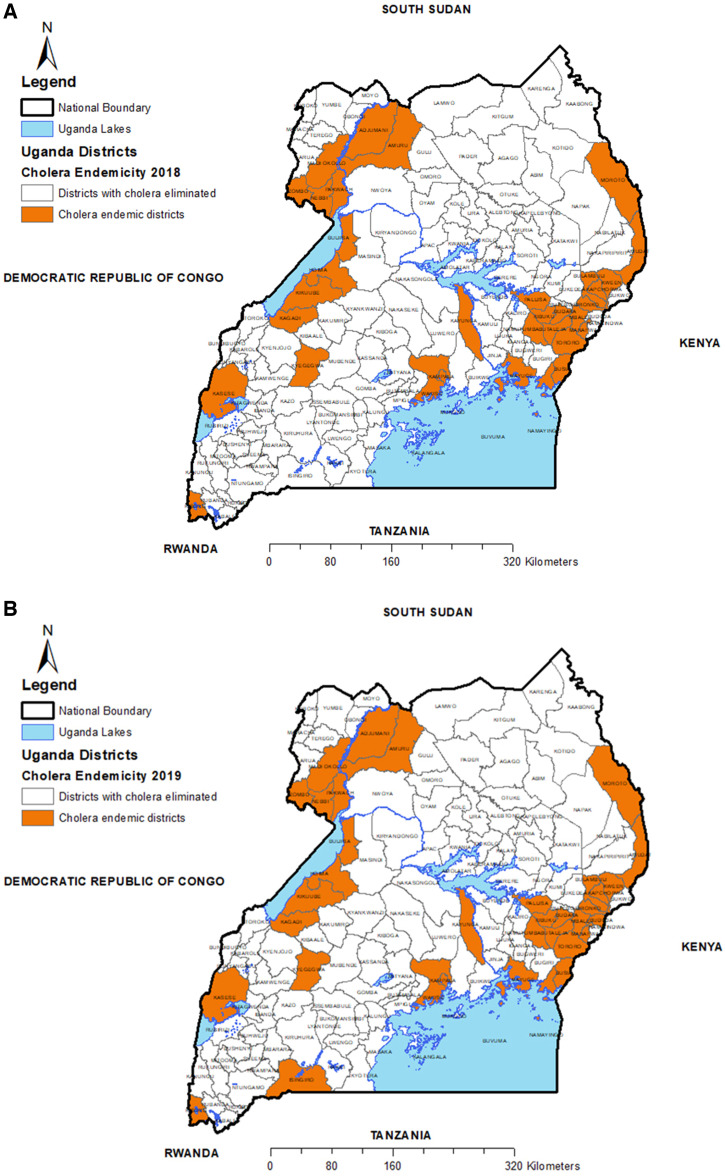

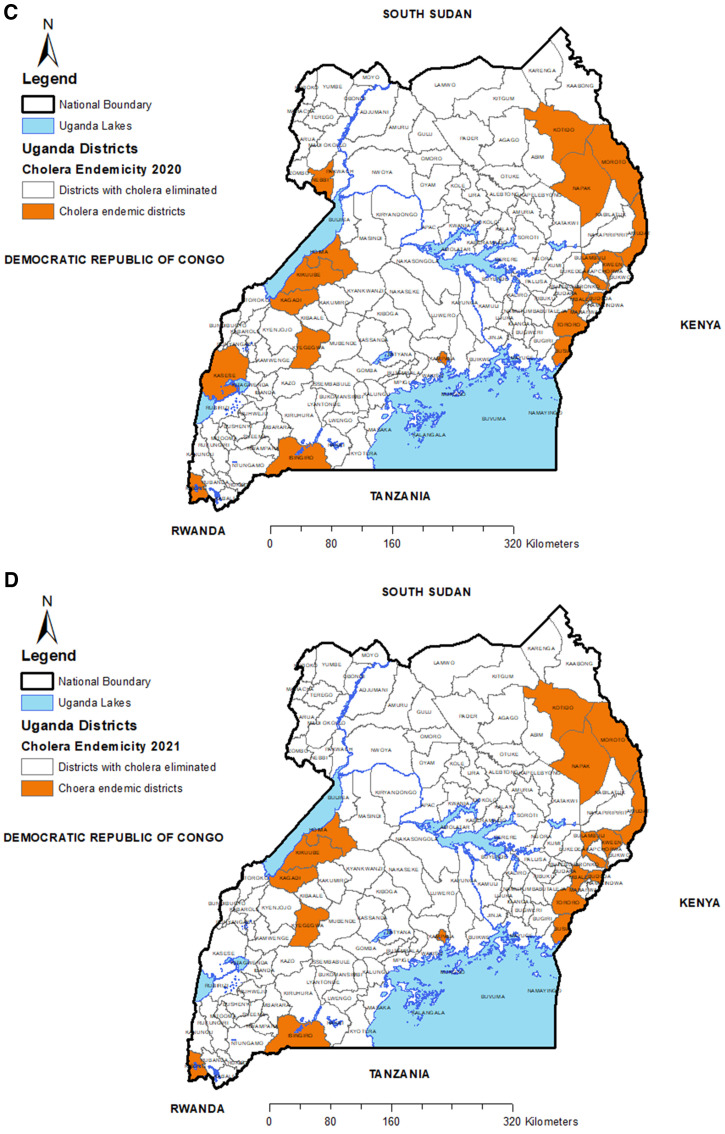
Uganda maps showing districts deemed to be endemic (shaded districts) and those deemed to have eliminated cholera (non-shaded districts) for (**A**) 2018, (**B**) 2019, (**C**) 2020, and (**D**) 2021.

Figure 4 shows the districts that were considered endemic during each year between 2018 through 2021. (Although the database included results from 2015, at least 4 years of data were needed to declare a district as having eliminated cholera.) The number of districts deemed to be endemic declined during this period from 36 districts in 2018 to 18 districts in 2021.

**Figure 5.**
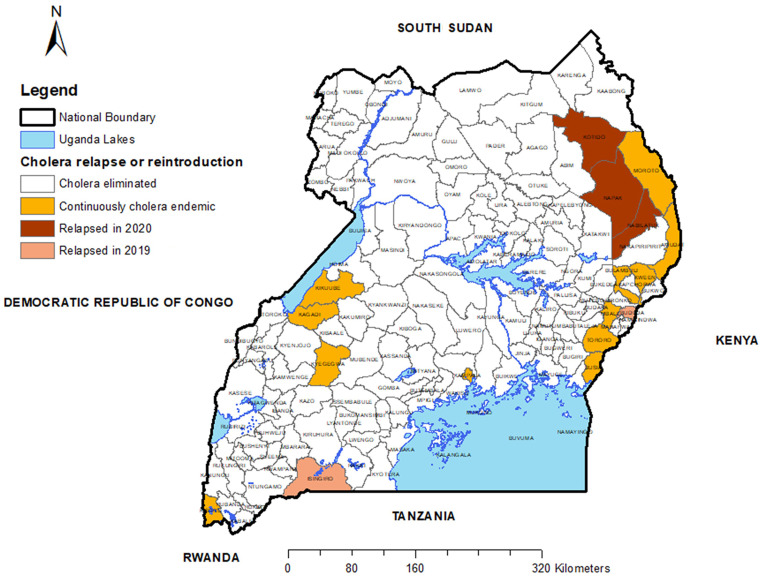
Uganda map showing districts that were continuously endemic 2015–2021 and those that had relapsed.

Figure 5 shows the districts that were continuously endemic during years 2018–2021, those that had eliminated cholera by 2021, and those that were deemed to have eliminated cholera but then relapsed. Notably, 13 districts continued to be endemic through this period, and five districts had relapsed.

## DISCUSSION

This study showed that the scorecard clearly illustrates the progress made by Uganda in reducing the number of districts with endemic cholera. During the period 2018–2021, the number of districts considered endemic declined by 50% (i.e., from 36 in 2018 to 18 in 2021). Based on reports from 2022 to date, we think that this number is likely to be reduced further in 2022. The reduction of endemic districts was especially remarkable in the districts that received vaccine in the western part of the country. These districts along the great lakes were previously identified as cholera hotspots and were consequently targeted for OCV campaigns. Although other factors may have contributed to this reduction in cholera in these districts, it seems vaccination played a major role in enhancing this reduction. In addition to vaccine, we will need to understand what other factors may have led to successful elimination in these formerly endemic districts; for example, did the vaccination campaigns lead to other behavioral changes that may have reduced the risk of transmission, or were there changes in the movement of refugees across the border from The Democratic Republic of the Congo or South Sudan?^20^ During the period 2020–2021, Uganda, like many other countries in Africa, was affected by COVID-19, and measures for COVID-19 control, such as lockdowns, promotion of WaSH, and health education, might also have had some impact on cholera prevention.^21^

The scorecard also identified “relapse districts,” which were districts that had apparently eliminated cholera but then developed outbreaks again. Because cholera was apparently gone from these districts, it seems likely that a new strain of *V. cholerae* was introduced into that district and that the district was clearly susceptible to its spread. Alternatively, the strain might have resided in the environment or might have been circulating among asymptomatic patients. Genomic data of these strains will help to determine mechanisms for relapse. Understanding the factors that led to the relapse will be important to understand how other districts that had eliminated cholera can prevent such a relapse and to determine how to eliminate cholera in these districts again.

One of the relapse districts in southern Uganda is one that housed refugees from the Democratic Republic of the Congo. As suggested in a recent paper,^20^ additional efforts will be needed to provide services (OCV and WaSH) to refugees as they register and before they move on to the refugee camp areas. In addition, more intensive WaSH and preventive vaccine interventions may be needed in these districts that have been designated for hosting refugees. This suggests that other criteria, in addition to being identified as a GTFCC hotspot, may be appropriate when determining where to use OCV. When designing such interventions, one must avoid stigmatizing refugees but should also be realistic about the vulnerabilities that accompany the migration of people from neighboring countries where cholera transmission is ongoing.

We found the scorecard method relatively easy to use. It did require accurately recording cases of cholera by district and subcounty, but it did not require complex computing techniques. In comparison to the GTFCC hotspot method, the scorecard adapts more rapidly to changing epidemiological situations. The GTFCC method uses data collected over several years (generally 5 years), but when OCV campaigns are carried out, the epidemiological patterns may change rapidly, as occurred in Uganda. When we applied the GTFCC method over a 7-year period, it continued to identify districts that were previously high-risk districts but did not continue to have high rates. When we compared the recent GTFCC hotspot map with the earlier hotspot map that used SaTScan,^16^ most of the same districts were identified as being high risk and would have been identified as ones to target for OCV.

An analysis of the outbreaks in Uganda suggests that most outbreaks are relatively short-lived. Although a few continue for many months, the median length of an outbreak was only 7.50 ± 1.66 weeks. When OCV is used reactively to control such outbreaks, there is little time to detect and characterize the outbreak, apply for vaccine, and implement the campaign before the outbreak has progressed to its downslope. On the other hand, if the country had a small national stockpile and could use it immediately, the vaccine may be more effective in controlling such an outbreak. In fact, the reactive vaccination campaigns in 2019 and 2020 (shown in Figure 2) were carried out using vaccine supplies that were left over from a previous preventive campaign.

This study benefitted by the availability of detailed surveillance data, which provided a line list of cases by date and district of origin, age, sex, and subcounty. It also benefited by a relatively small number of cases when compared with other countries, like The Democratic Republic of the Congo and Nigeria^22^^,^^23^ or Ethiopia,^24^ which, in some years, reports many thousands of cases. In Uganda, cholera occurs during defined outbreaks, and there does not appear to be year-round cholera in any area of the country.

The study has some limitations. The study depended on data from the routine surveillance system, and it is possible that the surveillance system did not detect some cases if they were rare or occurred outside of an outbreak. We feel that in Uganda, this is unlikely because of the awareness of the local health facilities to be alert to cholera symptoms. A second weakness is that the analysis did not identify cross-district spread of outbreaks but rather treated each district as if it was independent. Additional methods are needed to highlight transmission between districts as well as across national borders.^25^ Finally, the scorecard will benefit if it is coupled with rapid identification of the genomic lineage of the outbreak strains, enabling the tracking of the spread of outbreaks between countries and districts within the country.

The information from this analysis will provide additional evidence on which to develop the revised plan for cholera control and elimination to the year 2030. Given the progress being made, it does appear that Uganda is on track to meet the 2030 goal as described by the GTFCC.

## Financial Disclosure

This work was supported in part, by the Bill & Melinda Gates Foundation (OPP1148763; D. A. S.), a grant from the National Institute of Allergy and Infectious Disease (5R01AI123422; D. A. S.), and a grant from UNICEF AN# 43264783. The funders had no role in study design, data collection and analysis, decision to publish, or preparation of the manuscript.
